# Single Dose Intravenous Paracetamol versus Placebo in Postorthognathic Surgery Pain: A Randomized Clinical Trial

**DOI:** 10.1155/2024/8898553

**Published:** 2024-03-14

**Authors:** Thunshuda Sumphaongern, Pornchai Jansisyanont

**Affiliations:** Department of Oral and Maxillofacial Surgery, Faculty of Dentistry, Chulalongkorn University, Bangkok, Thailand

## Abstract

**Background:**

The postorthognathic surgery patients experienced moderate to severe pain and could be at risk for opioid-related side effects. The aim of this study was to evaluate the efficacy of a single dose of intravenous paracetamol to control postorthognathic surgery pain and reduce opioid consumption.

**Methods:**

The patients were randomized into two groups. The study group received intravenous paracetamol and the control group received a placebo immediately postoperation. The visual analogue pain scale (VAS) at 1-, 4-, 8-, 12-, 16-, 20-, and 24 -h postoperatively, morphine consumption, side effects from morphine, and patient satisfaction were analyzed.

**Results:**

Sixty-two patients (thirty-one patients in each group) were included. The postoperative VAS in the study group was significantly lower than those in the control group (*p* value <0.001) at all time points. The total postoperative morphine consumption in the study group (45.1 ± 21.2 mcg/kg) was significantly lower compared with the control group (136.5 ± 49.9 mcg/kg) (*p* value <0.001). Patient satisfaction was significantly higher in the study group (4.7 ± 0.5 out of 5 points) than in the control group (4.1 ± 0.7 out of 5 points) (*p* value <0.001). The incidence of nausea and vomiting was significantly lower in the study group compared with the control group (*p* value <0.001 and 0.002, respectively).

**Conclusion:**

A single dose of intravenous paracetamol as part of multimodal analgesia was effective for postorthognathic surgery pain. It provided significant benefits to patients, including reduced pain scores, decreased opioid consumption, reduced nausea and vomiting, and improved satisfaction. This trial is registered with TCTR20210908002.

## 1. Introduction

Orthognathic surgery is the surgical treatment for correcting the form, function, and abnormalities of the mandible and maxilla [1]. Most postorthognathic surgery patients experience postoperative pain and the pain had been assessed to be moderate to severe in intensity [2]. Orofacial pain has significant impacts on overall quality of life; including poor nutritional intake, uncomfortable communication, limited daily activities, mood disturbances, and compromised quality of sleep [3]. Inadequate pain control leads to physical function limitations, psychological distress, and poor quality of life and may evolve into chronic pain [4].

Many methods to manage postoperative pain in maxillofacial surgery have been introduced, including pharmacological and nonpharmacological methods [4]. Multimodal analgesia (MA) is the postoperative pain control strategy to use two or more analgesic modalities to achieve better pain control and to minimize the side effects that results in early recovery [5]. MA can include the using of opioids, nonopioid drugs, such as nonsteroid anti-inflammatory drugs (NSAIDs) and paracetamol, and adjuvant analgesics, with an additional neuraxial or regional nerve block [6].

Opioids are the medications used for the treating of acute and severe chronic pain, such as morphine, which is a popular opioid used for perioperative and acute postoperative pain after surgery. Although opioids provide good pain control, they have many adverse effects, namely, nausea, vomiting, constipation, urinary retention, pruritus, and respiration depression [7].

The use of anti-inflammatory medications, such as NSAIDs or corticosteroids can help reduce inflammation and pain after surgery. Administration of dexamethasone via intraoral submucosal or intramuscular route can reduce postoperative pain, swelling, and trismus after third molar surgery [8]. In addition, preoperative antibiotic prophylaxis can influence the postoperative inflammation and pain. It is still unclear whether administering antibiotics and the type of antibiotic formula have an impact on the pain after third molar extraction [9].

Paracetamol, known as acetaminophen in some countries, is one of nonopioids in MA. It has analgesic and antipyretic effects. The exact mechanisms of action of paracetamol are not fully understood; however, some evidence suggests that the proposed mechanisms of action are the effect in the hypothalamus for its antipyretic effect and the inhibition of cyclooxygenase (COX) activity for its analgesic effect [10]. However, paracetamol does not interfere with platelet function or have the anti-inflammation effect that NSAIDs have [11]. There are three routes for paracetamol administration, i.e., oral, rectal, and intravenous. Rectal paracetamol suppository has a slow onset and unpredictable bioavailability compared with the intravenous route and is more expensive compared with the oral route. Intravenous paracetamol has a faster onset of analgesia [12] and effectively reaches the plasma analgesic level [13] with greater peak plasma concentration [14] compared with the oral route at a similar dose. Moreover, intravenous paracetamol has more predictable pharmacodynamics and pharmacokinetics and less potential for hepatic injury. The disadvantage of intravenous paracetamol is due to the risk of intravenous cannulation, such as infection and thrombophlebitis [15]. The dosage recommendation for intravenous infusion is 1 g or 15 mg/kg in patients over 13 years old and adults who weigh less than 50 kg, every 6 h with a maximum dose of 4,000 mg per day [10]. Oral paracetamol is inexpensive and is simple to use [16]. Also, most patients are familiar with oral medications and readily accept using them. However, oral paracetamol is not appropriate for the immediate postoperative patient. Furthermore, most postorthognathic surgery patients have intermaxillary fixation (IMF) to stabilize the dental occlusion. Therefore, intravenous administration is the most appropriate route for acute postoperative orthognathic surgery pain control.

Paracetamol plays an important role in MA as one of the drugs that is used in postoperative pain management. MA is one of the main components of enhanced recovery after surgery (ERAS), which is a new concept regarding multimodal perioperative care to achieve rapid recovery after major surgical procedures. ERAS encourages practitioners to decrease the use of opioid analgesics (to reduce the drug's side effects and the length of stay) and use opioid-sparing methods, such as regional anesthesia, paracetamol, NSAIDs, and other analgesic adjuvant drugs, instead [5]. There are some studies regarding using an MA strategy in orthognathic surgery [17, 18] and the efficacy of intravenous paracetamol in orthognathic surgery and maxillofacial surgery pain control [19, 20]. The results seemed to be effective for the pain relief and decreasing opioid usage.

In addition, analgesic drugs cannot be given via the oral route in the immediate postoperative period and IMF may be used to stabilize the dental occlusion after postorthognathic surgery. Although the IMF helps to promote favorable dental occlusion by stabilizing the fractured segments, but it can also pose challenges in terms of maintaining airway patency [21]. Patients with IMF can be at risk for pulmonary aspiration, especially when nausea or vomiting occurs after general anesthesia or opioid administration.

Therefore, the purpose of this study was to evaluate the efficacy of a single dose of intravenous paracetamol to control postorthognathic surgery pain. The investigators hypothesized that the patients who received intravenous paracetamol will have better postoperative pain control, resulting in lower opioid-related side effects and higher satisfaction. The study's objectives were to evaluate the efficacy of a single dose of intravenous paracetamol for (1) the visual analogue pain scale (VAS) at first 24 h postoperatively; (2) the opioid consumption; (3) opioid-related side effects; and (4) the patients' satisfaction.

## 2. Materials and Methods

### 2.1. Ethics

This study was approved by the Human Research Ethics Committee of the Faculty of Dentistry, Chulalongkorn University (HREC-DCU 2020-101, on December 4, 2020) and was registered in the Thai clinical trials registry (TCTR20210908002). Informed consent was obtained from the patients before enrolling in the study. The study was performed in accordance with the Declaration of Helsinki.

### 2.2. Study Design and Population

A prospective, randomized clinical control study using 1 : 1 ratio of allocation was designed. Patients with age of 18–45 years old and American Society of Anesthesiology class I and II scheduled for bilateral sagittal split ramus osteotomy (BSSRO) surgery at the Faculty of Dentistry, Chulalongkorn University, from September 2021 to November 2022 were enrolled in this study. Patients with contraindications for intravenous paracetamol, such as a known allergy to paracetamol or to any of the other ingredients of the preparation or having severe hepatocellular insufficiency, allergy to morphine or NSAIDs, and incapable of communicating or using a visual analogue scale (VAS) or refused to participate in the study were excluded from the study.

### 2.3. Study Variables

The predictor variable was intravenous paracetamol, and the primary outcome was the VAS at every 4 hours in first 24 hours postoperatively. The secondary outcomes were morphine consumption, side effects from morphine, and patient satisfaction. Other study variables were the patient's demographics, types of BSSRO (advancement or setback), distance of BSSRO, operation time, blood loss, intraoperation opioid, types of prophylaxis antibiotics, presence of intermaxillary fixation, bad split, and nerve exposure.

### 2.4. Sample Size

The sample size was calculated using the formula for a randomized controlled trial [22] based on the visual analogue scale pain intensity from a previous study [19] (mean in the treatment group = 3.98, SD. in the treatment group = 0.69, mean in the control group = 4.63, SD. in the control group = 1.1, and ratio control/treatment = 1) with an alpha of 0.05 and 0.8 power, which indicated that at least 31 participants per group were required. Therefore, this study recruited 62 participants.

### 2.5. Data Collection

#### 2.5.1. Preoperation

The patients were randomized using block randomization (block size = 4) into two groups as follows: the study group received intravenous paracetamol and the control group received a placebo. The patients, the data collector, and postoperative care team were blinded to the group to which each patient had been assigned. The use of intravenous morphine patient-controlled analgesia (PCA) and the method for reporting the VAS pain scale for postoperative pain management were explained to the patients preoperatively. The VAS was scored 0–10 on a 10 cm line.

#### 2.5.2. Intraoperation

In both groups, general anesthesia was initiated by an anesthesiologist using intravenous propofol 2 mg/kg, fentanyl 1 mcg/kg, and atracurium 0.6 mg/kg. Nasotracheal intubation was inserted, and general anesthesia was maintained with desflurane, 50% nitrous oxide in oxygen, fentanyl, and atracurium by the same anesthesiologist for each patient. Ventilation was supported with targeted end-tidal carbon dioxide (EtCO2) ∼35 mmHg and their vital signs were monitored throughout the operations. Intravenous prophylaxis antibiotic drugs (penicillin G sodium 2 million units, cefazolin 1 g, clindamycin 600 mg, or amoxicillin-clavulanic acid 1.2 g) and 8 mg dexamethasone were administered. Two percent mepivacaine with adrenaline (1/100,000) 7.2 ml was used before the surgical incision to reduce blood loss. The BSSRO surgeries were performed by the same surgical team with the same standard technique. During the operation, intravenous fentanyl 25 mcg was administered as required every 10 minutes if the patient had elevated blood pressure and heart rate more than 15% from their baseline values. The last dose of fentanyl was administered at least 60 min prior to the end of the operation and the total amount of intraoperative fentanyl usage was recorded. Ondansetron (0.15 mg/kg) for preventing nausea and vomiting and 40 mg parecoxib was given intravenously before the operation was finished. After the operation was finished, extubation was performed.

#### 2.5.3. Postoperation

The drug and placebo were prepared by the investigator in advance and the postoperative care team did not know whether it was paracetamol or placebo. After immediate arrival at the recovery room, a single dose of intravenous 15 mg/kg paracetamol was administered over 15 minutes to the patients in the study group, whereas normal saline as the placebo was administered in a similar manner to the patients in the control group. Intravenous morphine PCA was prescribed to both groups. The morphine PCA setting was 1 mg bolus, no continuous dose, with a lock-out interval of 5 minutes and a 4-hour limit of 30 mg. No additional forms of paracetamol or NSAIDs were given in the first 24 h postoperatively. The VAS pain scale at 1-, 4-, 8-, 12-, 16-, 20-, and 24-h postoperatively were accessed by the data collector. Morphine consumption, side effects from morphine, and patients' satisfaction with the postoperative pain control were recorded using 5-point scale (0 = most unsatisfactory to 5 = most satisfactory) at 24-h postoperatively.

### 2.6. Statistical Analysis

The data were analyzed using IBM SPSS Statistics for Windows, version 22.0 (IBM, Armonk, New York, USA). The categorical characteristics are presented as frequencies and percentages. The differences between the treatment groups were analyzed using Pearson's chi-square test or Fisher's exact test for the categorical basic characteristics and the side effects from morphine. The continuous basic characteristics were tested for normality by the Shapiro–Wilk test and presented as means and standard deviations. The differences in each continuous characteristic between the treatment groups were analyzed using the independent *t*-test or the Mann–Whitney *U* test as appropriate. Because each primary and secondary outcome variable was correlated, multivariate analysis of variance (MANOVA) was used to compare the treatment effects simultaneously on these variables. A *p* value <0.05 was considered significant. The significant associations after Bonferroni multiple testing correction were *p* value <0.005, which was calculated from 0.05/9 (numbers of test).

## 3. Results

**Figure 1:**
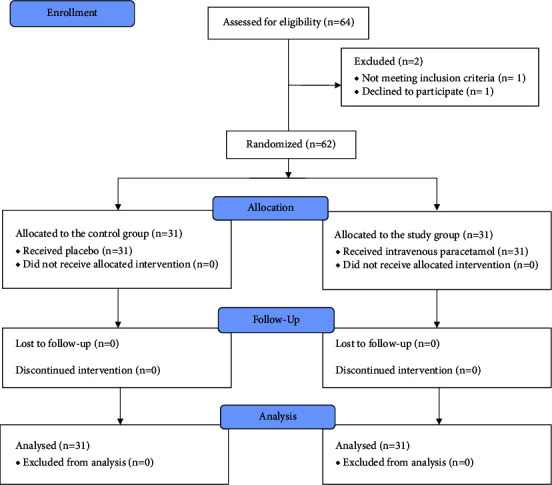
Flow diagram of the study.

Sixty-two patients, from September 2021 to November 2022, were included in this study. The flow diagram of the study is presented in Figure 1. The patients' age ranged from 18 to 44 years old. There were no significant differences in the treatment groups' characteristics, i.e., age, sex, weight, height, body mass index (BMI), types of BSSRO (advancement or setback), maximal BSSRO distance (the maximal distance of the adjustment of BSSRO), operation time, bleeding, amount of intraoperative fentanyl usage, postoperation with or without IMF, kinds of preoperative antibiotic prophylaxis, operation with complication of bad split, and the exposure of inferior alveolar nerve during the operation (*p* value>0.05) (Table 1).

**Figure 2:**
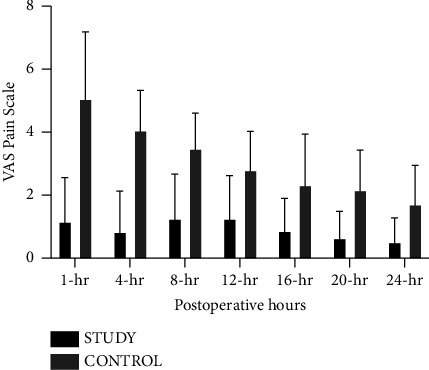
Distribution of the VAS pain scale.

**Figure 3:**
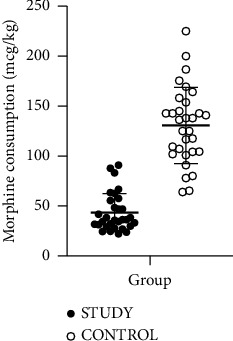
Distribution of morphine consumption.

The MANOVA results revealed a significant difference in outcome variables between the treatment groups (*p* < 0.001). The VAS pain scale, morphine consumption, and patients' satisfaction in pain control are presented in Table 2. The postoperative VAS pain scale at 1-, 4-, 8-, 12-, 16-, 20-, and 24-h postoperatively in the study group was significantly lower than those in the control group (*p* value <0.001) at each time point (Figure 2). After Bonferroni multiple testing correction, the VAS pain scale in the study group remained significantly lower than in the control group at each time point. For total postoperative morphine consumption, the study group demonstrated significantly lower morphine consumption than in the control group (*p* value <0.001) (Figure 3). There was significantly greater satisfaction in the study group than in the control group (*p* value <0.001).

The side effects from morphine, such as respiratory depression, vomiting, nausea, pruritus, and urinary retention, were observed in all patients. As illustrated in Table 3, there were significantly fewer incidents of nausea and vomiting in the study group than in the control group (*p* value <0.001 and 0.002, respectively). Furthermore, the incidence of pruritus and respiratory depression was lower in the study group than in control group; however, this was not significantly different (*p* value =0.053 and 1.000, respectively). No patient complained about urine retention in this study.

## 4. Discussion

The results of the present study demonstrated that the postoperative orthognathic surgery pain intensity was moderate at 1- and 4 h postoperative in the control group by administering opioid analgesics and parecoxib for analgesia. The study group's pain intensity was mild and significantly decreased at each time point during the first 24 h postoperative by administering a single dose of intravenous paracetamol in conjunction with opioid analgesics and parecoxib. The previous literature mentioned that postoperative orthognathic surgery patients reported moderate to severe pain and had high opioid consumption. Furthermore, patients who underwent mandibular surgery reported higher pain and required more analgesic drugs than those who underwent only maxillary surgery [23]. A single dose of intravenous paracetamol could relief the pain after surgery for mandibular fractures [19]. It has been reported that there was a similar efficacy between a single dose of 1 g intravenous paracetamol and 75 mg intramuscular diclofenac sodium in postoperative pain control for orthognathic surgery [24]. In addition, a systematic review presented that the combined use of paracetamol and NSAIDs for acute postoperative pain had better analgesia than the use of either drug alone [25]. Corticosteroids, as adjuvant drugs, synergize the analgesia and reduce postoperative swelling in maxillofacial surgery [4]. These results supported that the combination of opioids (fentanyl and morphine), paracetamol, NSAIDs (parecoxib), and dexamethasone, which was prescribed based on the MA and ERAS concepts for postoperative pain analgesia in this study, was effective and could significantly decrease the postoperative orthognathic surgery pain, especially in the study group.

In the present study, the total postoperative morphine consumption significantly decreased by 66.8% after administering the single dose of intravenous paracetamol when combined with the analgesic effect of NSAIDs and dexamethasone. Similarly, intravenous paracetamol was shown to effectively reduce opioid consumption after orthognathic surgery [20] and decreased the morphine requirement by 33% over 24 h after major orthopedic surgery [26]. As reported in a systematic review and meta-analysis, a single dose of 1 g intravenous paracetamol reduced pain up to 50% in 37% of patients and decreased the opioid requirement by 26% over 4 h compared with placebo [27].

The patients' satisfaction was significantly higher in the intravenous paracetamol group compared with the control group, which was consistent with another trial [15]. However, a previous trial found that intravenous paracetamol did not reduce the opioid-related side effects [28]. In contrast with the present study, the study group had a significantly lower incidence of nausea and vomiting compared with the placebo group. These results might be due to the marked decrease in opioid analgesics due to the synergistic effect of multimodal analgesic drugs administration.

The strength of this study was the pain control method in postorthognathic surgery that follow the MA and ERAS protocol, resulting in better patient's welfare and satisfaction. Whereas the weakness and limitations of this study were the long average operation time and some of the surgeons in our institute still applied IMF routinely in every case although there were no differences in the sample of the study and the control group. Future studies might be the opioid-free analgesia by application of the multiple doses of intravenous paracetamol combined with other nonopioid drugs in the MA manner for acute postoperative pain control.

## 5. Conclusion

A single dose of intravenous paracetamol as part of multimodal analgesia was effective for postorthognathic surgery pain. It provided significant benefits to patients, including reduced postoperative first 24 hours' pain scores, decreased opioid consumption, reduced postoperative nausea and vomiting, and improved patient satisfaction. However, other opioids-related side effects, such as pruritus, urinary retention, and respiratory depression were not significantly different when compared with the placebo.

## Figures and Tables

**Table 1:** Characteristics of the study participants according to the treatment groups.

Characteristics	Total (*n* = 62)	Study group (*n* = 31)	Control group (*n* = 31)	*p* value
Age (year), mean ± SD	25.8 ± 5.3	25 ± 5.2	26.6 ± 5.4	0.105^a^
Sex, *n* (%)				0.054^c^
Male	19 (30.6)	6 (19.4)	13 (41.9)	
Female	43 (69.4)	25 (80.6)	18 (58.1)	
Weight (kg), mean ± SD	62.6 ± 12.2	62.3 ± 11	62.9 ± 13.4	0.851^b^
Height (cm), mean ± SD	166.9 ± 7.6	165.2 ± 7.7	168.5 ± 7.3	0.092^b^
BMI, mean ± SD	22.4 ± 3.6	22.8 ± 3.3	22 ± 3.8	0.301^a^
BSSRO type, *n* (%)				1.000^d^
Advancement	5 (8.1)	2 (6.5)	3 (9.7)	
Setback	57 (91.9)	29 (93.5)	28 (90.3)	
Maximal BSSRO distance (mm), mean ± SD	5.8 ± 2	5.7 ± 2	6 ± 2	0.539^a^
Operation time (min), mean ± SD	237.5 ± 48.7	240.2 ± 44.2	234.8 ± 53.4	0.665^b^
Blood loss (ml), mean ± SD	365.3 ± 162.6	356.5 ± 156.4	374.2 ± 170.7	0.750^a^
Intraoperative fentanyl (mcg/kg), mean ± SD	1.9 ± 0.5	1.9 ± 0.5	1.8 ± 0.4	0.773^a^
Intermaxillary fixation (IMF), *n* (%)				0.796^c^
With IMF	25 (40.3)	13 (41.9)	12 (38.7)	
Without IMF	37 (59.7)	18 (58.1)	19 (61.3)	
Antibiotics, *n* (%)				0.095^c^
Penicillin G sodium	51 (82.3)	27 (87.1)	24 (77.4)	
Cefazolin	5 (8.1)	3 (9.7)	2 (6.5)	
Clindamycin	5 (8.1)	0 (0)	5 (16.1)	
Amoxicillin-clavulanic acid	1 (1.6)	1 (3.2)	0 (0)	
Bad split, *n* (%)				0.492^d^
Yes	2 (3.2)	0 (0)	2 (6.5)	
No	60 (96.8)	31 (100)	29 (93.5)	
Exposed inferior alveolar nerve, *n* (%)				1.000^c^
Yes	22 (35.5)	11 (35.5)	11 (35.5)	
No	40 (64.5)	20 (64.5)	20 (64.5)	

^a^Differences between treatment groups were tested using the Mann–Whitney *U* test. ^b^Differences between treatment groups were tested using the independent *t*-test. ^c^Differences between treatment groups were tested using Pearson's chi-square test. ^d^Differences between treatment groups were tested using Fisher's exact test.

**Table 2:** Pain scores, morphine consumption, and patients' satisfaction according to the treatment groups.

Characteristics	Study group (*n* = 31)	Control group (*n* = 31)	*p* value^*∗*^
VAS pain scale, mean ± SD			
1 hour	1.2 ± 1.4	5.1 ± 2.1	**<0.001**
4 hour	0.8 ± 1.3	4.2 ± 1.4	**<0.001**
8 hour	1.3 ± 1.4	3.5 ± 1.1	**<0.001**
12 hour	1.3 ± 1.4	2.9 ± 1.3	**<0.001**
16-hour	1.0 ± 1.1	2.3 ± 1.6	**<0.001**
20-hour	0.7 ± 0.9	2.3 ± 1.4	**<0.001**
24-hour	0.6 ± 0.9	1.8 ± 1.3	**<0.001**
Morphine consumption (mcg/kg), mean ± SD	45.1 ± 21.2	136.5 ± 49.9	**<0.001**
Satisfaction (0–5), mean ± SD	4.7 ± 0.5	4.1 ± 0.7	**<0.001**

^*∗*^Differences between treatment groups were tested using MANOVA. A significant difference after Bonferroni multiple testing correction (*p*<0.05) is indicated in bold.

**Table 3:** Side effects from morphine according to the treatment groups.

Characteristics	Total (*n* = 62)	Study group (*n* = 31)	Control group (*n* = 31)	*p* value
Nausea, *n* (%)				**<0.001** ^a^
Yes	14 (22.6)	1 (3.2)	13 (41.9)	
No	48 (77.4)	30 (96.8)	18 (58.1)	
Vomit, *n* (%)				**0.002** ^b^
Yes	9 (14.5)	0 (0)	9 (29)	
No	53 (85.5)	31 (100)	22 (71)	
Pruritus, *n* (%)				0.053^b^
Yes	5 (8.1)	0 (0)	5 (16.1)	
No	57 (91.9)	31 (100)	26 (83.9)	
Urine retention, *n* (%)				1.000^b^
Yes	0 (0)	0 (0)	0 (0)	
No	62 (100)	31 (100)	31 (100)	
Respiratory depression, *n* (%)				1.000^b^
Yes	1 (1.6)	0 (0)	1 (3.2)	
No	61 (98.4)	31 (100)	30 (96.8)	

^a^Differences between treatment groups were tested using Pearson's chi-square test. ^b^Differences between treatment groups were tested using Fisher's exact test. A significant difference (*p* < 0.05) is indicated in bold.

## Data Availability

The data used to support the findings of this study are included within the article.

## Additional Points

This trial is registered with Thai clinical trials registry (TCTR20210908002).
